# The distribution of aerobic bacteria in Chinese cropland is linked to the soil texture

**DOI:** 10.3389/fmicb.2025.1541460

**Published:** 2025-02-19

**Authors:** Xiao Jiamin, Wu Haonan, Xu Chao, Hu Yajun, Xu Zhiwen, Zhang Dongxu, Li Abo, Wei Xiaomeng, Ge Tida, Wei Gehong

**Affiliations:** ^1^State Key Laboratory for Crop Stress Resistance and High-Efficiency Production and Shaanxi Key Laboratory of Agricultural and Environment Microbiology, College of Life Sciences, Northwest A&F University, Xianyang, China; ^2^College of Natural Resources and Environment, Northwest A&F University, Xianyang, China; ^3^Key Laboratory of Agro-Ecological Processes in Subtropical Region, Institute of Subtropical Agriculture, The Chinese Academy of Sciences, Changsha, China; ^4^Leading Bio-Agricultural Co., Ltd., Qinhuangdao, Hebei, China; ^5^Horticulture Institute, Ningxia Academy of Agriculture and Forestry Sciences, Yinchuan, China; ^6^State Key Laboratory for Quality and Safety of Agro-Products, International Science and Technology Cooperation Base for the Regulation of Soil Biological Functions and One Health of Zhejiang Province, Ningbo University, Ningbo, China

**Keywords:** aerobic bacteria, *gltA* gene, soil texture, water management patterns, community composition

## Abstract

Aerobic bacteria extensively drive the carbon cycle in soil owing to their vigorous respiration; however, their geographical distribution and mechanisms remain poorly understood. The citric acid synthetase-encoding gene (*gltA*), which encodes the key enzyme in the tricarboxylic acid cycle of aerobic respiration, was used as a marker gene to investigate the geographical distribution of aerobic bacteria in Chinese agricultural fields. The abundance and diversity of *gltA*-harboring bacteria changed unimodally as the latitude increased, with peak values at middle latitudes, where the dominant species showed the lowest relative abundance. Despite the different water management practices, our data found little difference in the abundance, diversity, or relative abundance of the dominant species of *gltA*-harboring bacteria between paddy and upland soils on a large scale, which was significantly affected by the soil type (black, fluvo-aquic, and red), which can be defined by the soil texture. Linear regression and random forest model analyses indicated that soil texture strongly regulated the community of *gltA*-harboring bacteria, particularly the abundance of this functional guild. Generally, less abundant and diverse *gltA*-harboring bacteria were observed in soils with higher clay content. We identified biomarkers in the different soil types using linear discriminant analysis effect size analysis. The results suggest a significant correlation between soil texture and most of these biomarkers. Additionally, the biomarkers in black soil were mainly *r*-strategists, which include *Proteobacteria, Actinobacteria*, and *Bacteroidetes*, were positively correlated with soil organic carbon content. In contrast, the biomarkers in fluvo-aquic soil were generally *K*-strategists, such as *Acidobacteria, Ktedonobacteraceae, Planctobacteriaceae*, and *Frankia* were negatively correlated with soil organic carbon content. These different biomarkers likely play distinct roles in soil carbon sequestration. This study provides foundational insights into the role of aerobic bacteria in soil and enhances our understanding of microbial contributions to the biogeochemical cycle of carbon.

## Introduction

Microorganisms catalyze almost all biogeochemical cycles in soil. Based on the electron acceptors used to produce energy, microorganisms are classified as either aerobic or anaerobic. Anaerobic bacteria play critical roles in many soil processes, such as denitrification, sulfur reduction, and iron redox reactions, and their functional groups have been extensively studied (Täumer et al., 2022; Hülse et al., 2021; Wang et al., 2022). In contrast, the aerobic bacterial community in agricultural soils remains largely unexplored. For carbon (C) metabolism, the rate of aerobic respiration is 15–20 times higher than that of anaerobic respiration (Fairbairn et al., 2023). In the topsoil of agricultural fields, intensive tillage creates better air permeability than other land use types, which ensures high O_2_ availability. Therefore, aerobic microorganisms strongly contribute to C mineralization in agricultural soil, which is among the most active carbon emission streams (Stochmann et al., 2013; Liang et al., 2017). Compared to natural ecosystems, the average soil respiration rates in agricultural soil are 96%−386% higher, leading to 25%−75% lower soil organic carbon (SOC) content (Lai et al., 2012; Barba et al., 2018; Huang et al., 2021). Because of its substantial role in determining soil fertility and health, the sequestration and maintenance of SOC have long been a core issue in studies focusing on agricultural soils (Lehmann et al., 2020).

The tricarboxylic acid (TCA) cycle is the key pathway for organic matter decomposition during the aerobic respiration of biota, including microorganisms. Citrate synthase catalyzes the first and most essential steps of the TCA cycle. As previously reported, the citrate synthase-encoding gene (*gltA*) is universally distributed across the life tree and is a single copy in the bacterial kingdom, with few horizontal gene transformations (Castro et al., 2012). In addition, the phylogenetic relationship of *gltA* is distinct from that of eukaryotes (Schnarrenberger and Martin, 2002). Owing to these advantages, *gltA* has been used as a marker gene to detect environmental aerobic bacteria (Siles and Margesin, 2018; Wang et al., 2023). Castro et al. (2012) designed a primer set targeting bacterial *gltA* and validated it in multiple soils with high amplification efficiency by real-time quantitative PCR and the recovery of major bacterial groups identified by 16S rRNA gene sequencing of the same sample (Castro et al., 2012; Siles and Margesin, 2018). These studies provide an opportunity to study the abundance and diversity of aerobic bacteria in agricultural soil.

The texture of soil critically regulates air permeability and, therefore, O_2_ concentration (Bronick and Lal, 2005; Lu et al., 2023), which critically constrains the community and activity of aerobic microorganisms (Zibiske and Bradford, 2007; Abdul Rahman et al., 2021). In addition, soil texture determines the pore-based transportation of water and nutrients (Pei et al., 2021; Veloso et al., 2023) and provides microscale heterogeneity of soil which is necessary for niche separation and co-occurrence of diverse microorganisms (Curd et al., 2018; Yang et al., 2024). The major croplands in China are distributed in the Northeast Plain, North China Plain, and lower-middle reaches of the Yangtze River, which account for more than 78% of Chinese cereal production. Because of variations in parent material and regional climate, the soil texture gradient along latitudes in China shows a transition from coarse-textured soils in the Northeast and North China plains to finer-textured soils in the lower-middle reaches of the Yangtze River (Sheng et al., 2015). In agricultural fields, water management is a major practice that affects air permeability in the soil matrix. As the flooding condition in paddies creates anoxic ambient soil, while the unsaturated water content in uplands allows the intrusion of abundant O_2_, there are likely wide differences in the community of aerobic microbiota in Chinese cropland soil; however, this is largely unexplored.

Therefore, using *gltA* as a marker gene, this study investigated the spatial distribution pattern and main driving factors of aerobic bacteria. Amplicon sequencing was performed on 88 soil samples from three main soil types (black, fluvo-aquic, and red soil) and two water management modes (paddy and upland) spanning a large area. The abundance and diversity of *gltA*-harboring bacteria were examined and their correlations with soil texture, pH, SOC, mean annual precipitation (MAP), and mean annual temperature (MAT) were determined. Specifically, this study aimed to (1) determine whether the abundance of *gltA*-harboring bacteria differs between paddy soil and upland soil and (2) assess how the community composition of *gltA*-harboring bacteria differs by soil type.

## Materials and methods

### Soil sampling and pretreatment

A total of 88 soil samples were collected from agricultural fields spanning the main cereal-producing areas of China, including 36 black soils in Northeast China, 22 fluvo-aquic soils in the North China Plain, and 30 red soils in southern China (Supplementary Figure S1). Considering the major crop in each area, the soils were planted by the major crop in each area during the sampling time, which was summer maize in Northeast China and North China Plain, while paddy rice in South China. Mean annual precipitation (MAP) and mean annual temperature (MAT) of the sampling sites were obtained from the China Meteorological Data Service Center (https://data.cma.cn/en).

Five 1 m × 1 m sampling squares were assigned to each site in a plot of 20 m × 20 m. Three topsoil cores (0 cm−20 cm) were randomly collected from each sampling square during the crop growth season (June–July 2017). All the soil cores from the same sampling site (5 × 3 = 15) were combined into a single sample and transported to the laboratory on ice within 48 h. The fresh soils were sieved through a 2 mm sieve after the rocks and plant residues were removed by hand. To minimize the effects of weather, agricultural management, and plant growth, the soils were incubated for 2 weeks at 25°C under controlled water conditions in the laboratory (upland soils were adjusted to 45% water-holding capacity and paddy soils were flooded using the deionized water with a 2 cm water layer). After incubation, soil samples were harvested and divided into two fractions: one fraction was frozen in liquid nitrogen and stored at −80°C for DNA extraction and the other was air dried for the analyses of soil physiochemical properties.

### Soil physiochemical property analyses

The soil texture was analyzed using the fixed pipette method (Dane and Topp, 2002). SOC was determined using the heat K_2_Cr_7_O_2_-H_2_SO_4_ titration method (Bao, 2000). The soil total nitrogen content was determined using the Kjeldahl method (Bremner, 1965). Soil-available phosphorus (AP) was extracted using 0.5 M NaHCO_3_ and determined using the ammonium molybdate method (Olsen, 1954). Soil pH was determined using a pH meter (Mettler Toledo, Zurich, Switzerland) at a water-to-soil ratio of 1:2.5.

### DNA extraction

Total soil DNA was extracted using a Mobio PowerSoil DNA kit (Mo Bio Laboratories, Inc., Carlsbad, CA, USA) according to the manufacturer's instructions. DNA quality and concentration were quantified using a NanoDrop spectrophotometer (NanoDrop Technologies; Wilmington, DE, USA) and via the electrophoresis on a 1% agarose gel. The isolated DNA was stored at −20°C for subsequent analyses.

### Real-time quantitative PCR

Real-time quantitative PCR analysis was performed to determine the abundance of aerobic bacteria using *gltA* as a marker gene. The primers used were CS680F (5′AYG CCG ABC AYG ARY WSA A-3′) and CS904R (5′TAS ACS SGR TGR CCR AAG CCC AT) (Castro et al., 2012). The PCR system included 5 μL of 2 × SYBR PreMix ExTag (Takara, Japan), 1 μL each of forward and reverse primers (10 mmol/L), 1 μL of DNA template (diluted to 10 ng/μL), and 2 μL of ddH_2_O. The PCR conditions were as follows: 95°C for 5 min, followed by 40 cycles of 95°C for 30 s, 56°C for 30 s, and 72°C for 30 s. The fluorescence signal was collected at the end of each cycle. A standard curve was prepared by 10-fold gradient dilution of plasmids containing the target fragment and was considered validated when *R*^2^ > 0.98, with an amplification efficiency of 90%−110%.

### Amplicon sequencing and reads processing

The *gltA* gene from each sample was amplified using the CS680F/CS904R primer set and specific barcodes. The PCR system included 12.5 μL of 2 × PreMix Ex Taq (Takara, Japan), 2 μL of each forward and reverse primer, 1 μL of DNA template (diluted to 25 ng/μL), and 7.5 μL of ddH_2_O. The PCR conditions were as follows: 95°C for 5 min; 30 cycles of 95°C for 30 s, 56°C for 30 s, and 72°C for 30 s; and a final extension at 72°C for 10 min. PCR products were purified using a Tiangen gel extraction kit (Tiangen, Beijing, China) and sequenced on an Illumina HiSeq PE2500 platform (Illumina, San Diego, CA, USA).

Pair-end raw reads were assembled, screened, and trimmed using QIIME (Caporaso et al., 2010). Low-quality tags were removed as well as those that were < 180 bp or more than 250 bp. The clean sequences were exposed to a chimera filter and frameshift correction in the RDP Fungene pipeline (Fish et al., 2013), and subsequently clustered into OTUs at 97% similarity. Only OTUs affiliated with more than two sequences observed in more than two samples were retained. A representative sequence was selected for each OTU, and BLAST against the GenBank non-redundant nucleotide database was used for taxonomic assignment. All datasets were normalized to the same sequencing depth (20,000 sequences per sample) for subsequent analyses. Raw reads were deposited in the NCBI Sequence Read Archive under the accession number PRJNA1067286.

### Statistical analysis

All statistical analyses were performed using R (v4.0.2; http://www.r-project.org/), except for the kriging interpolation algorithm. Ordinary kriging interpolation in ArcGIS 10.7 software was used to draw the spatial distributions of the abundance and richness of *gltA*-harboring bacteria. OTUs richness calculation and PCA analysis were performed using the estimateR and *rda* function “vegan” package, respectively (Oksanen et al., 2022). Differences in abundance and richness of *gltA*-harboring bacteria communities in the different group sequences were calculated using the Kruskal-Wallis test in the “EasyStat” package (Wen, 2020). Permutational multivariate analysis of variance (PERMANOVA) was used to evaluate significant differences between the groups. Both the dissimilarity matrix of the *gltA*-harboring bacteria community and soil texture were calculated using the vegdist function in the “vegan” package (Oksanen et al., 2022), and linearized to calculate the relationship between the two indices.

To evaluate the relative importance of environmental factors on the *gltA* abundance, richness, and community composition of *gltA*-harboring bacteria, a random forest model was performed using the “rfPermute” packages (Archer, 2022). Linear discriminant analysis (LDA) effect size (LEfSe; Segata et al., 2011) was performed using the Galaxy online pipeline (http://huttenhower.sph.harvard.edu/galaxy/) to discover marker taxa in different soil types using default parameters.

## Results

### Geographical distribution of *gltA-*harboring bacterial community

**Figure 1:**
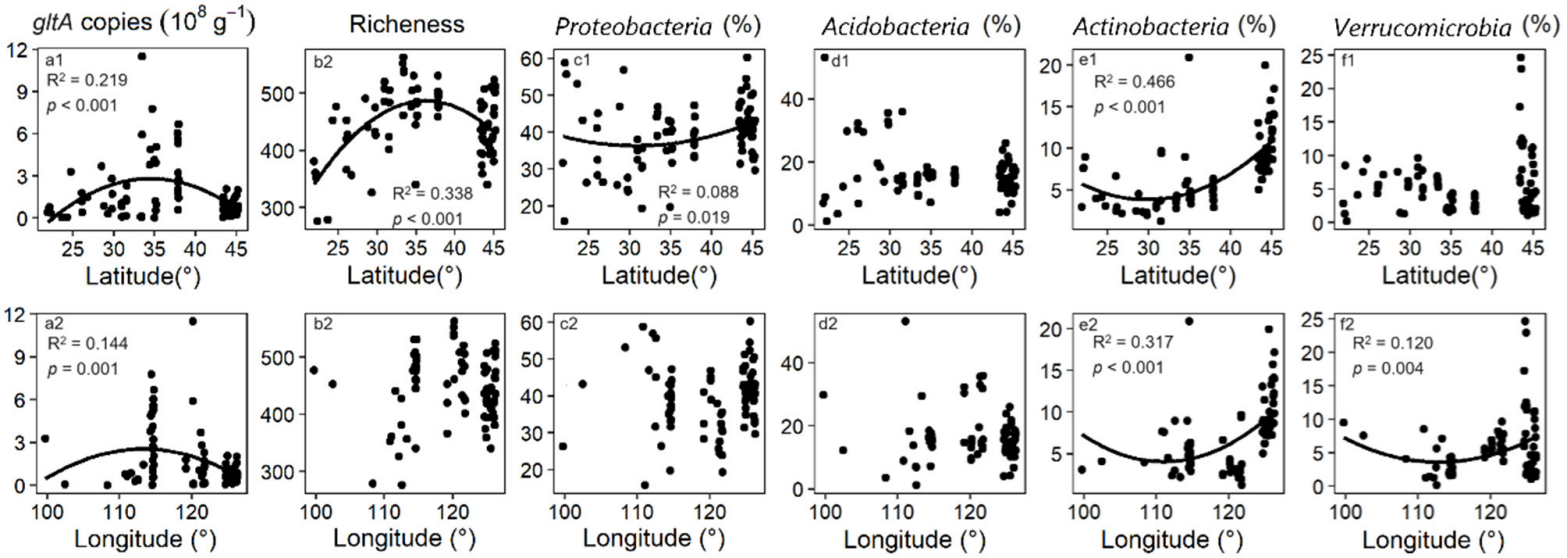
Quadratic regression of *gltA* abundance, richness and first 4 phyla of *gltA*-harboring bacterial community to the latitude **(a1–f1)** and longitude **(a2–f2)**. Insignificant regression curves are not shown (*p* < 0.05).

The zonal distribution of the abundance (indicated by *gltA* copy number) and diversity (indicated by *gltA* richness) of *gltA*-harboring bacteria was revealed using Kriging interpolation (Supplementary Figure S2). The *gltA* abundance and diversity in the samples ranged from 1.3 × 10^7^-1.2 × 10^10^ g^−1^ and 276–563, respectively (Supplementary Figures S2A, B), and unimodally changed with increasing latitude, with both peaking at the middle latitude of ~35° (N) (Figures 1a1, b1). A similar trend was observed for *gltA* abundance with increasing longitude but not for *gltA* diversity (Figures 1a2, b2). Species annotation detected 1144 *gltA*-harboring OTUs affiliated with 36 phyla (Supplementary Figure S3). On average, *Proteobacteria, Acidobacteria, Actinobacteria*, and *Verrucomicrobia*, accounted for 68% of the total reads in all samples. However, these phyla were less dominant in the central region than in the northern or southern regions (Supplementary Figure S3). As shown in Figures 1c1–f1, c2–f2, *Proteobacteria* and *Actinobacteria* presented a latitude-associated zonal distribution with the highest relative abundance at 31°-32° (N), whereas a longitude-associated zonal distribution was found for *Actinobacteria* and *Verrucomicrobia*, with the highest relative abundance at ~113° (E).

### Effect of soil texture and land use on the community of *gltA-*harboring bacteria

**Figure 2:**
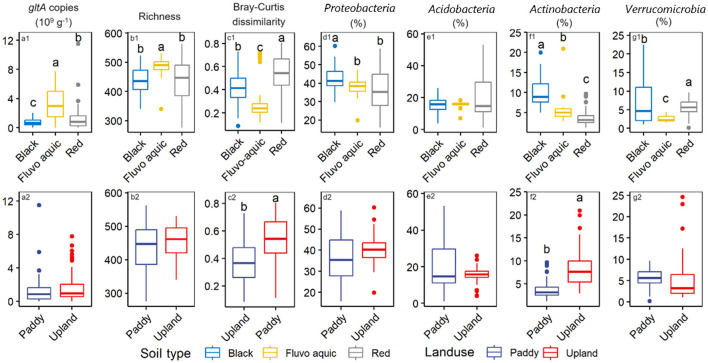
Effect of soil type and landuse on the abundance **(a1, a2)**, richness **(b1, b2)**, the community composition as indicated by the Bray-Curtis dissimilarity of *gltA*-harboring bacterial community **(c1, c2)**, and first 4 phyla of *gltA*-harboring bacterial community **(d1–g1, d2–g2)**. Different letters above the boxes indicate significant difference (*p* < 0.05).

Soil type remarkably affected *gltA* abundance, with the highest content in fluvo-aquic soil (3.3 × 10^9^ copies g^−1^), followed by red soils (1.5 × 10^9^ copies g^−1^), and the lowest content in black (0.8 × 10^9^ copies g^−1^) (Figure 2a1, *p* < 0.05). Similar results were observed for *gltA* diversity, with no significant differences between the red and black soils (Figure 2b1). However, a comparison between paddy and upland soils revealed no significant difference in either the abundance or diversity of *gltA* (Figures 2a2, b2). Bray-Cutis dissimilarity indicated significant differences in the overall community composition of *gltA*-harboring bacteria among the soil types, as well as between upland and paddy soils (Figures 2c1, c2). Nevertheless, PCoA and PERMANOVA analyses revealed a significant effect of soil type on *gltA*-harboring bacterial communities (Supplementary Figure S4, *R*^2^ = 0.432, *p* = 0.001). Additionally, soil type affected the relative abundance of the dominant phyla more strongly than land use. As shown in Figures 2c1–f1, significant differences among the soil types were observed for all four dominant phyla, except *Acidobacteria*. Generally, *Proteobacteria* and *Actinobacteria* were more abundant in the black soil, whereas *Verrucomibia* was more abundant in the red soil. In contrast, difference in the relative abundance of the dominant phyla between the upland and paddy soils, except more abundant *Actinobacteria* in paddy soils than in upland soils (Figures 2c2–f2).

### Impact of soil texture on *gltA*-harboring bacterial community

**Figure 3:**
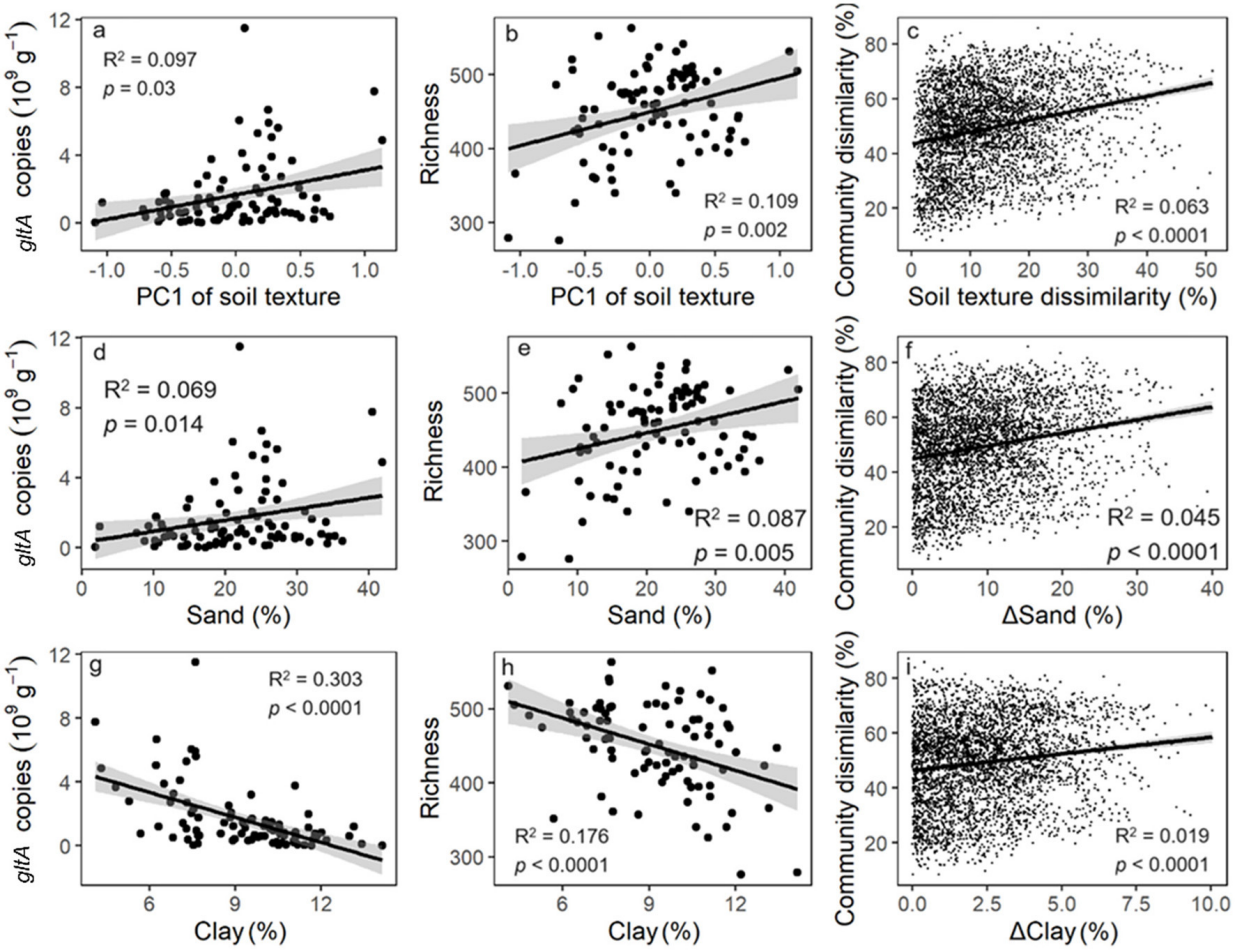
Relationship of *gltA* abundance **(a, d, g)**, richness **(b, e, h)** and community composition **(c, f, i)** to the soil texture which is represented by the first axis of principal component analysis on the soil aggregate composition and the content of sand, silt and clay in the soil. The relationships to the silt content are insignificant and was not shown.

**Figure 4:**
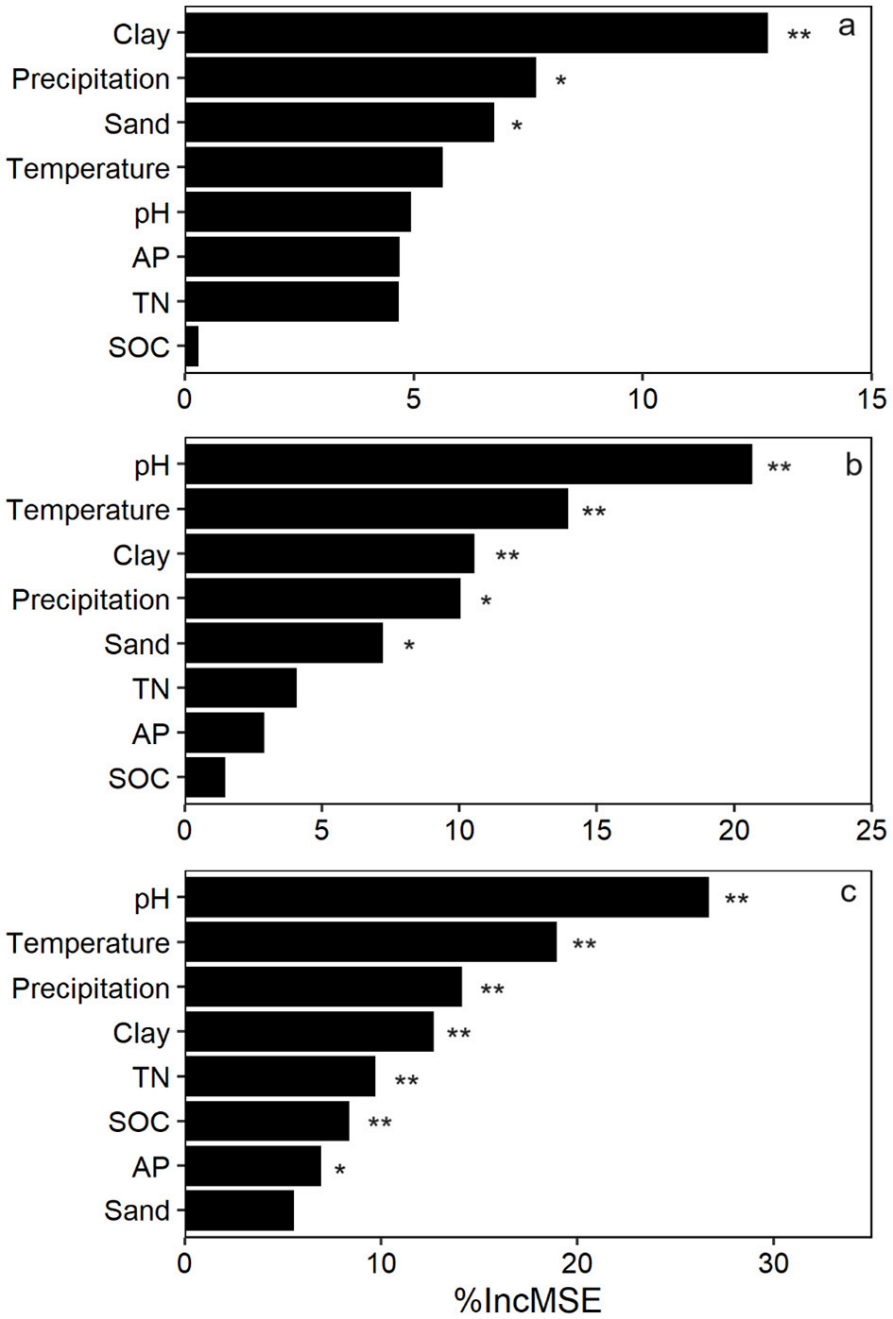
Random forest models revealing the effect of environmental factors on the *gltA* abundance **(a)**, richness **(b)**, and community composition of *gltA*-harboring bacteria **(c)**. Asterisks at the right of the bars indicate significant effect of the variations: *p < 0.05; **p < 0.01.

*gltA* copy number and richness increased linearly along the PC1 axis of soil texture (Figures 3a, b). Similarly, a significant positive correlation was observed between the Bray and Curtis distance of *gltA*-harboring bacterial community and soil texture (Figure 3c). These results indicated that soil texture was tightly correlated with the community of *gltA-*harboring bacteria. Further analysis revealed that the abundance, diversity, and community composition of *gltA*-harboring bacteria were most closely related to clay content, followed by sand, but not to silt content (Figures 3d–i). In particular, both the copy number and richness of *gltA* significantly increased with the relative clay content (*R*^2^ = 0.176–0.303, *p* < 0.0001), but decreased with that of sand (*R*^2^ = 0.069–0.087, *p* < 0.05). The random forest model explained 34.0%, 36.1%, and 81.5% of the variation in *gltA* abundance (Figure 4a), richness (Figure 4b), and community composition of *gltA*-harboring bacteria (Figure 4c), respectively. Among all climatic and soil properties, clay content was the most important predictor of *gltA* abundance, followed by annual precipitation and sand content (*p* < 0.01). Similarly, clay content was a significant predictor of the richness and community composition of *gltA*-harboring bacteria (Figures 4b, c, *p* < 0.01).

### Biomarkers of *gltA*-harboring bacteria in different soil types

**Figure 5:**
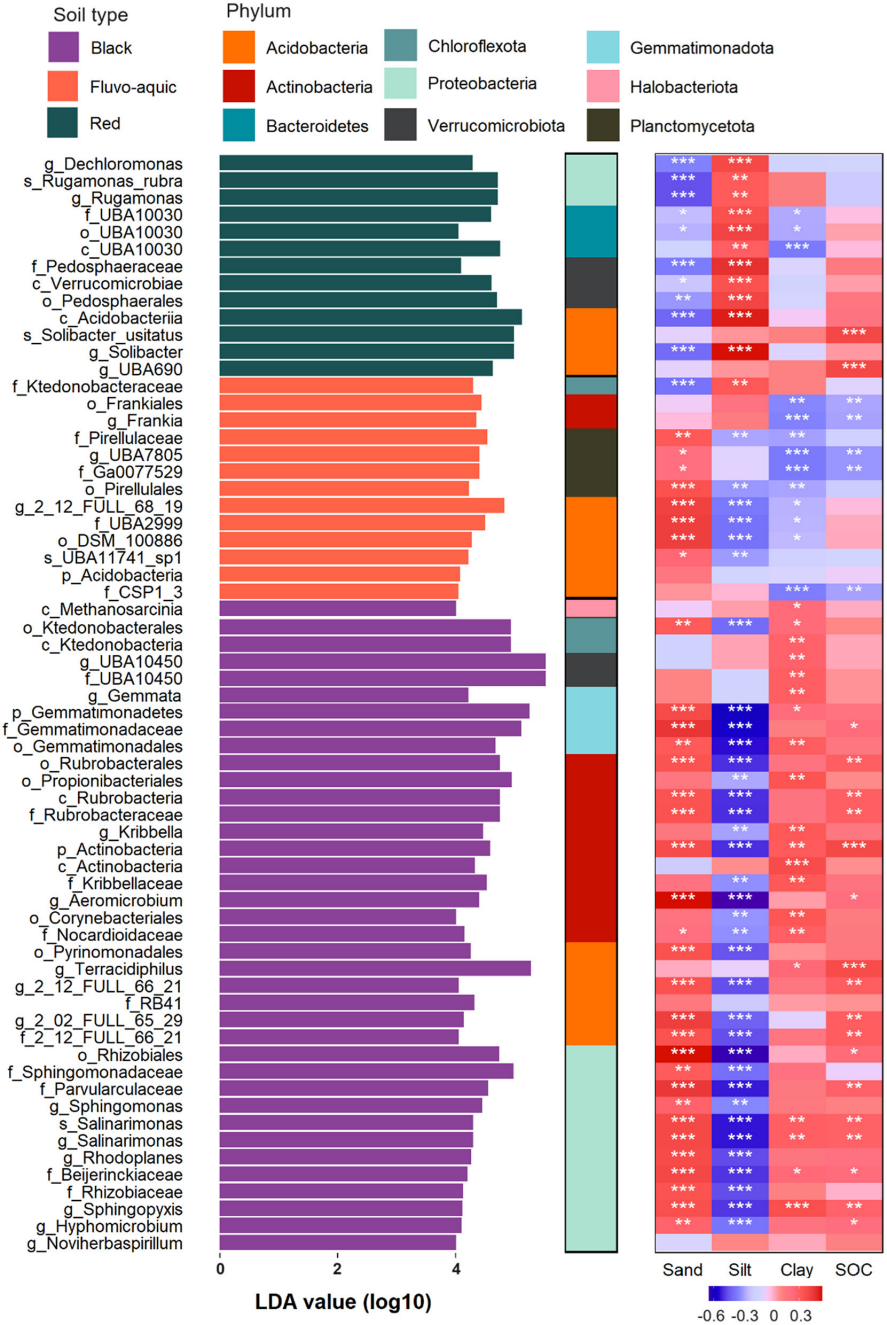
Biomarkers of *gltA*-harboring bacteria in different soils revealed by LEfSe analysis and their relationship to soil texture and SOC content. Significant correlations are indicated by the asterisks (*p < 0.05; **p < 0.01; ***p < 0.001).

LEfSe analysis revealed that 39, 13, and 13 biomarkers were enriched in black, fluvo-aquic, and red soils, respectively (Figure 5). Most of the *gltA*-harboring bacteria enrichen in black soil and were affiliated with *Proteobacteria* (30.8%) and *Actinobacteria* (28.2%), followed by *Acidobacteria* (15.4%) and *Gemmatimonadetes* (10.3%). *Planctomycetota* and *Bateroidetes* were uniquely enriched in fluvo-aquic and red soils, respectively. The enrichment of *Verrucomicrobiota* was also found in red (23.1%) and black (5.1%) soils, but not in fluvo-aquic soil. The relative abundances of the biomarkers were strongly correlated with the contents of the different soil aggregate fractions (Figure 5). Generally, the biomarkers in black soil were positively correlated with the contents of sand and clay but negatively correlated with silt, whereas the biomarkers in fluvo-aquic soil were negatively correlated with clay and silt but positively correlated with sand, and the biomarkers in red soil were positively and negatively correlated with silt and sand.

## Discussion

The highest abundance and diversity of aerobic bacteria were observed in the middle latitudes (Figures 1a1, b1). This is consistent with the results of 16S rRNA gene sequencing (Delgado-Baquerizo et al., 2018; Kerfahi et al., 2024; Xia et al., 2020), which suggests a uniform geographical distribution of aerobic bacteria within the general bacterial community (Liu F. et al., 2020; Zhang et al., 2020). Despite having no significant effect on aerobic bacterial diversity, the *gltA* copy number was hump-shaped by longitude (Figure 1a2). As soil texture has been identified as the primary regulator of the abundance of aerobic bacteria, the above phenomenon is caused by zonal variation in soil texture from the coast to the interior (Liu S. et al., 2020). Nevertheless, the relative abundance of the dominant phyla valley-shape varied with the latitude (Figures 1c1–f1). The results suggested that the dynamics of aerobic bacterial abundance and diversity as affected by latitude were mainly attributed to the flourishing or decline of rare species.

Owing to distinct water management practices, discrepant microbial communities are expected between soils from paddy fields and uplands, especially for biota groups that require special redox conditions. However, it is surprising that our data found a similar abundance and alpha diversity of aerobic bacteria was found in the paddy and upland soils (Figure 2). Although bacterial communities have been extensively studied in both upland and paddy soils, they have rarely been compared in a single study, and no consistent conclusions have been reached. Lee et al. (2020) found higher bacterial richness in paddy soils than in upland soils. However, contrasting results were reported by Li et al. (2024). As the bacterial community can rapidly respond to plant growth, fertilization, precipitation, and other natural or anthropogenic disturbances, unlike the direct extraction of DNA from fresh soil immediately frozen after sampling, this study pre-incubated the soils to minimize these transient effects. Our data suggest that the contradictory results of previous studies are likely due to differences in field management and other short-term factors. Generally, paddy fields are exposed to the wetting-drying cycles during the rice season and remain dry after harvest during the fallow (Johnson et al., 2024; Bo et al., 2024). Therefore, the redox conditions between paddy and upland areas might be less disparate, as expected at the annual scale, leading to a minimum effect on the abundance and diversity of aerobic bacteria. Nevertheless, the paddy and upland soil was sampled from south and north China, respectively, which might induce factors to cover up the effect of the landuse types. Therefore, we suggest geographically balanced sampling in future studies.

Instead of land use, soil type significantly influenced the aerobic bacterial community (Figure 2). This was strongly correlated with differences in soil texture (Figures 3, 4). Soil texture is a key predictor of soil aeration and water retention and previous studies have indicated that it is an important factor in shaping soil microbial communities (Xia et al., 2020). Our data revealed that the abundance and diversity of aerobic microorganisms were significantly and positively correlated with the sand particle content in the soil (Figure 3). This could be because the higher the proportion of sand particles in the soil, the greater the oxygen content in the soil pores. Studies have shown that large aggregates contain a substantial amount of easily accessible carbon and energy sources for microbes (Hemkemeyer et al., 2018; Karimi et al., 2018). However, an increase in sand content is often accompanied by a decrease in soil water retention and an increase in resource heterogeneity, which increases bacterial diversity.

The biomarkers in the black soil were mainly *r*-strategists, including *Proteobacteria, Actinobacteria*, and *Bacteroidetes*. This is likely due to the perfect fertility of this soil type (Wang et al., 2024). Most of these biomarkers were significantly and positively correlated with SOC content, suggesting their contribution to soil C sequestration (Figure 5). Biomarkers in black and fluvo-aquic soils were positively correlated with sand content, probably because a higher sand proportion in the soil text is beneficial for air permeability in upland soils and provides more ecological niches by improving soil heterogeneity (Xia et al., 2020; Hemkemeyer et al., 2018; Karimi et al., 2018). In contrast, the biomarkers in fluvo-aquic soil were *Acidobacteria, Ktedonobacteraceae, Frankia*, and *Planctobacteriaceae*, which were generally *K*-strategists with low carbon use efficiency (Fierer et al., 2007; Yang et al., 2022). Therefore, the negative correlation between the biomarkers and SOC in the fluvo-aquic soil likely indicates that the high respiration but low biomass production of these bacteria induced a net loss of SOC. In addition, we found filamentous bacteria and extracellular polymer producers in the biomarkers (*Ktedonobacteraceae, Frankia*, and *Planctobacteriaceae*) of the fluvo-aquic soil (Chang et al., 2011; Bhattacharyya et al., 2024; Fuerst and Sagulenko, 2011), which drive the aggregation of clay and silt into large soil particles (Olagoke et al., 2022). This may explain the negative correlation between clay and silt content and biomarkers (Figure 5). We found no relationship between the biomarkers and SOC in the red paddy soil, probably because of the more complex C cycling mechanism. The root exudates and straw input to the paddy soil are incompletely decomposed during the rice season, with recalcitrant plant residues further stabilized by aerobic bacteria during the fallow period when the soil is dry (Chen et al., 2021). Biomarkers in red soil are recognized for their ability to degrade cellulose, hemicellulose, and aromatic compounds, suggesting that they mainly take part in the latter stage of the C cycle (Yang et al., 2021; Austin and Moss, 1986; Challacombe et al., 2011) and are not adequate to explain C sequestration in paddy soils.

## Data Availability

The datasets presented in this study can be found in online repositories. The names of the repository/repositories and accession number(s) can be found in the article/Supplementary material.
