# Supplementary material for: The distribution of aerobic bacteria in Chinese cropland is linked to the soil texture
Source: Front Microbiol. 2025 Feb 19;16:1541460. doi: 10.3389/fmicb.2025.1541460 (PMC11880006; doi:10.3389/fmicb.2025.1541460)

Figure S1: Spatial distribution of sampling points. Shape of the dots represents different soil types, and different colors represent different land use modes.


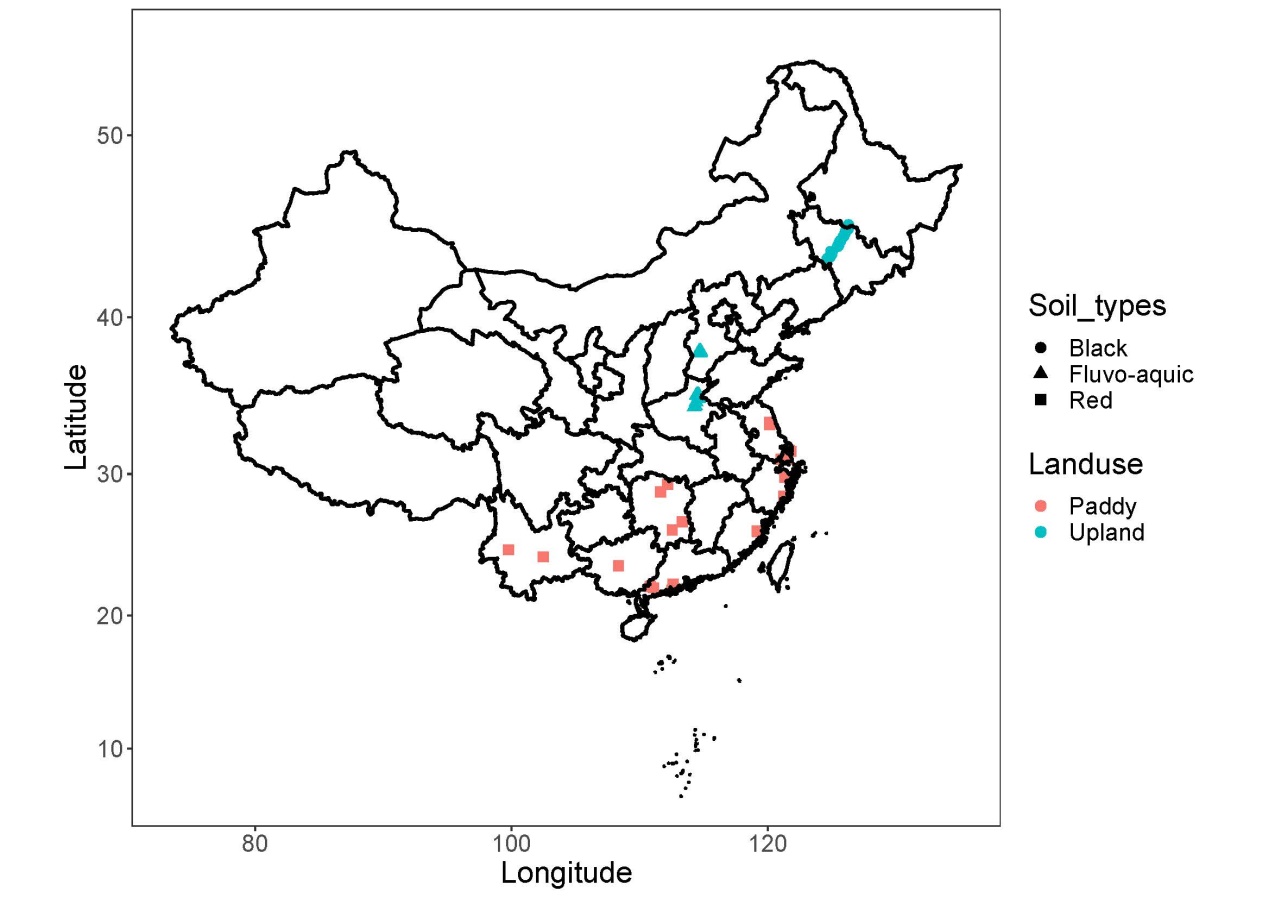


Figure S2. General distribution patterns of *gltA*-harboring bacteria abundance (A) and richness of gltA-harboring bacteria (B). Spatial distributions were mapped by kriging interpolation.


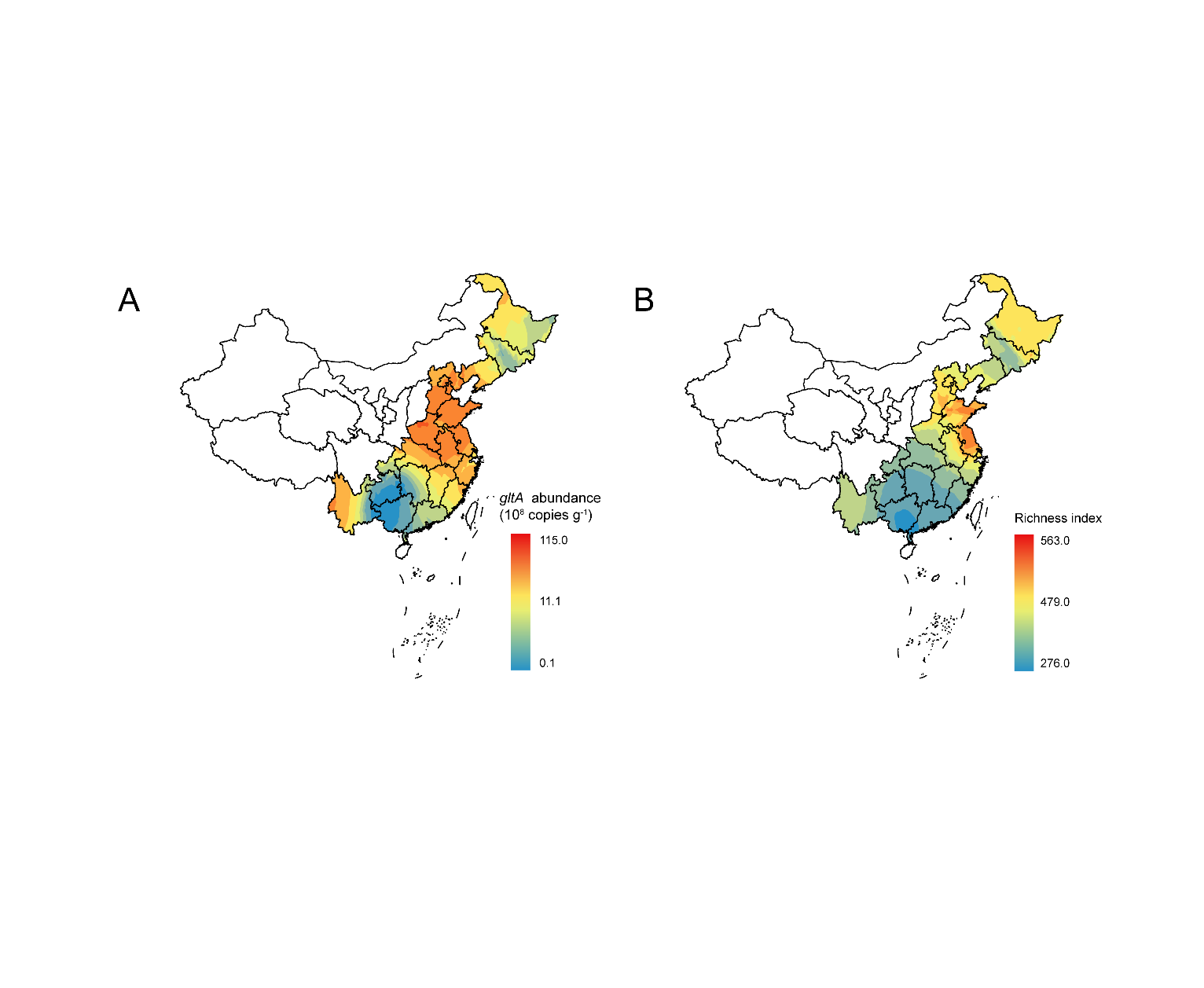


Figure S3. Phylum-level distribution of the *gltA*-harboring bacteria.


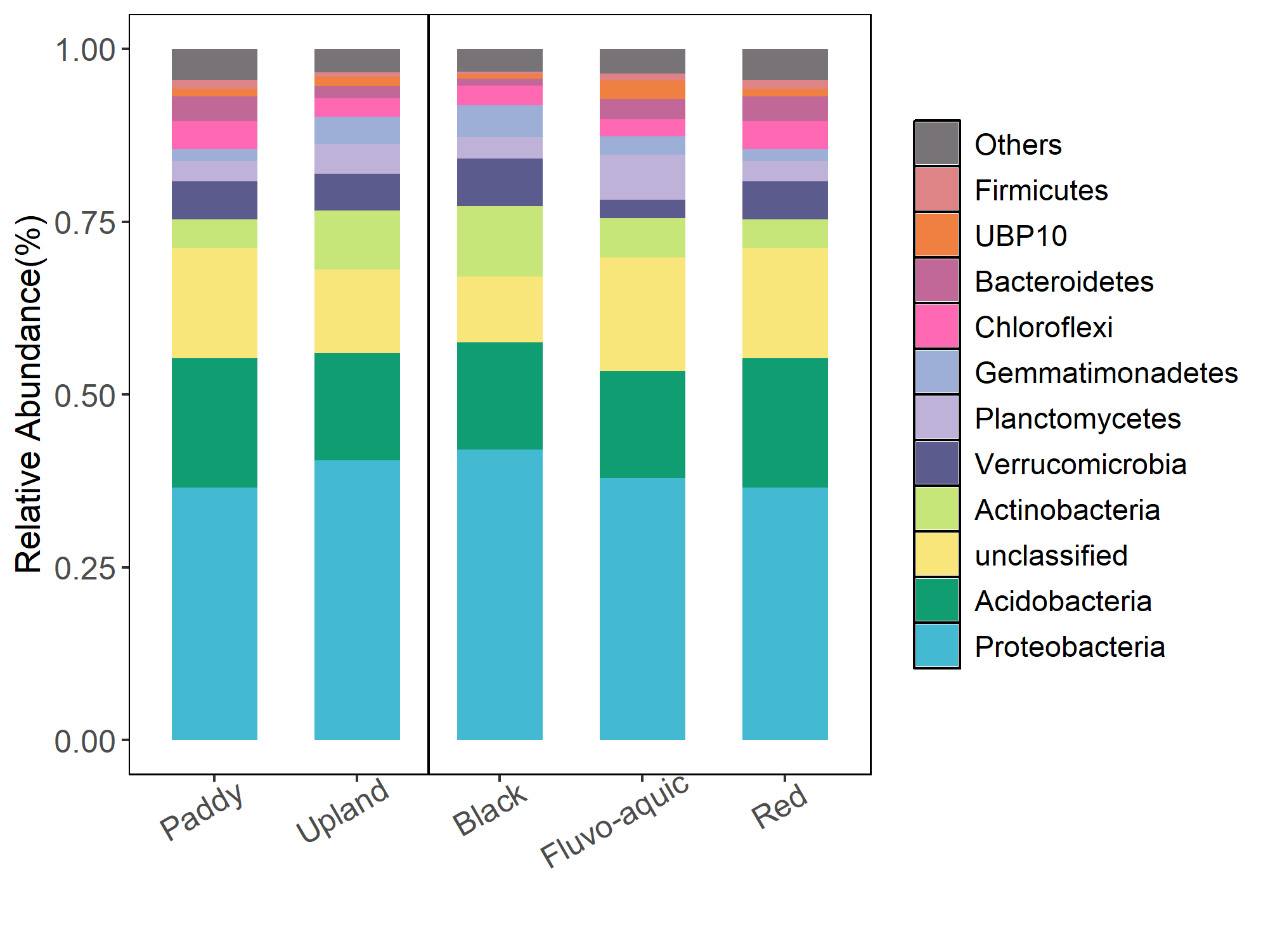


Figure S4. PCoA and PEMANOVA analyses based on soil types as variables.


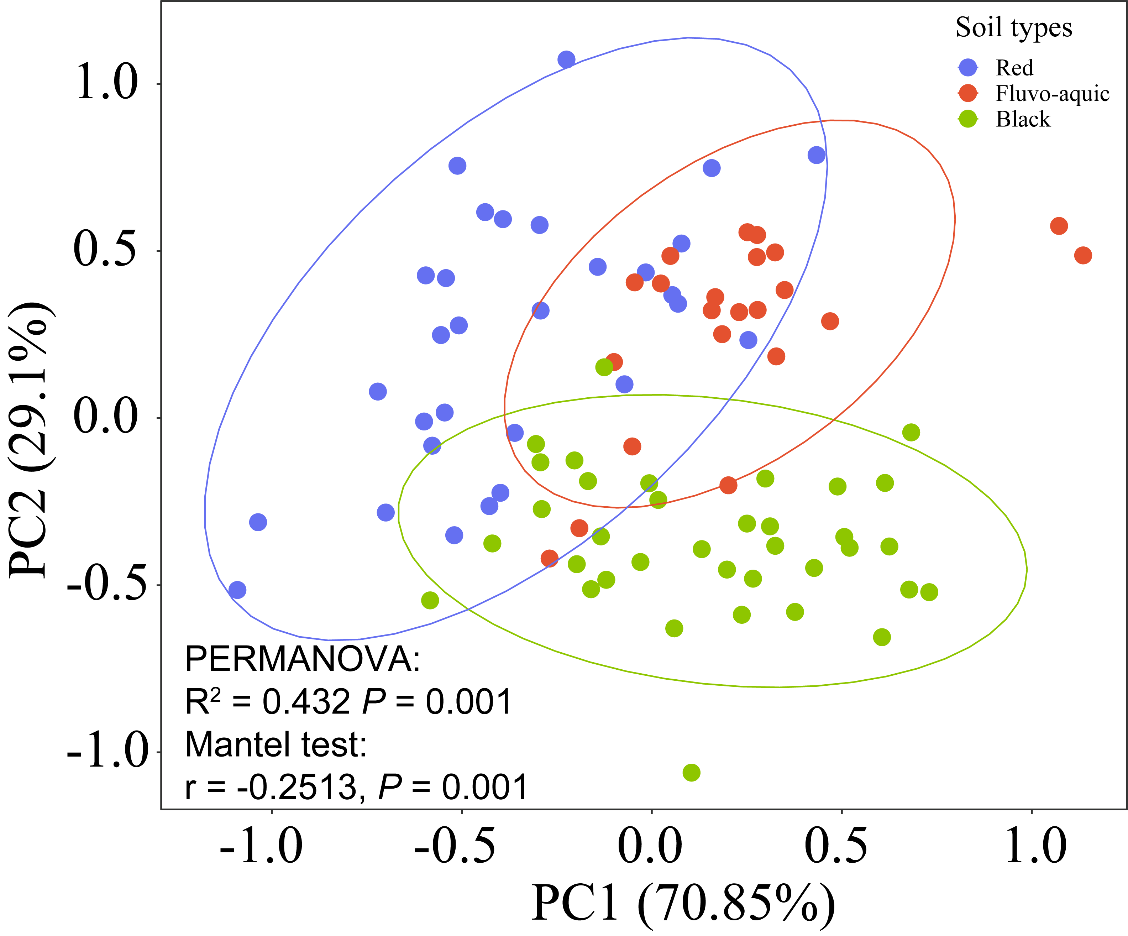

Supplement: Supplementary file 3 [file Data_Sheet_3.docx]
